# Field evaluation of a point-of-care triage test for active tuberculosis (TriageTB)

**DOI:** 10.1186/s12879-023-08342-5

**Published:** 2023-07-03

**Authors:** Tracy R. Richardson, Bronwyn Smith, Stephanus T. Malherbe, Jane Alexandra Shaw, Firdows Noor, Candice MacDonald, Gian D. van der Spuy, Kim Stanley, Alida Carstens, Tarryn-Lee Fisher, Ilana van Rensburg, Marika Flinn, Candice Snyders, Isaac Johnson, Bernadine Fransman, Hazel Dockrell, Guy Thwaites, Nguyen Thuy Thuong Thuong, Claudia Schacht, Harriet Mayanja-Kizza, Mary Nsereko, Elisa M. Tjon Kon Fat, Paul L.A.M. Corstjens, Annemieke Geluk, Morton Ruhwald, Adam Penn-Nicholson, Novel N. Chegou, Jayne Sutherland, Gerhard Walzl, TrENDx consortium, Andriëtte Hiemstra, Susanne Tonsing, Gerard Tromp, Muyiwa Owolabi, Joseph Mendy, Awa Gindeh, Amadou Barry, Georgetta Mbayo, Julia Buech, Malte Streitz, Sophie Nalukwago, Ann Ritah Namuganga, Dorcas Lamunu, Michael Odie, Louise Pierneef, Anouk van Hooij, Morten Ruhwald, John Belisle, Karen Dobos, Mark Hatherill, Thomas Scriba, Jill Winter

**Affiliations:** 1 Stellenbosch University, Cape Town, South Africa; 2 London School of Hygiene and Tropical Medicine, London, UK; 3 University of Oxford, Oxford, UK; 4 LINQ Management, Berlin, Germany; 5 Makerere University, Kampala, Uganda; 6 Leiden University Medical Centre, Leiden, The Netherlands; 7 Foundation for Innovative New Diagnostics, Geneva, Switzerland; 8 London School of Hygiene and Tropical Medicine, Banjul, The Gambia

**Keywords:** TB, Tuberculosis, Biomarker, Biosignature, Non-sputum based screening

## Abstract

**Background:**

To improve tuberculosis (TB) diagnosis, the World Health Organisation (WHO) has called for a non-sputum based triage test to focus TB testing on people with a high likelihood of having active pulmonary tuberculosis (TB). Various host or pathogen biomarker-based testing devices are in design stage and require validity assessment. Host biomarkers have shown promise to accurately rule out active TB, but further research is required to determine generalisability. The TriageTB diagnostic test study aims to assess the accuracy of diagnostic test candidates, as well as field-test, finalise the design and biomarker signature, and validate a point-of-care multi-biomarker test (MBT).

**Methods:**

This observational diagnostic study will evaluate sensitivity and specificity of biomarker-based diagnostic candidates including the MBT and Xpert® TB Fingerstick cartridge compared with a gold-standard composite TB outcome classification defined by symptoms, sputum GeneXpert® Ultra, smear and culture, radiological features, response to TB therapy and presence of an alternative diagnosis. The study will be conducted in research sites in South Africa, Uganda, The Gambia and Vietnam which all have high TB prevalence. The two-phase design allows for finalisation of the MBT in Phase 1 in which candidate host proteins will be evaluated on stored serum from Asia, South Africa and South America and on fingerstick blood from 50 newly recruited participants per site. The MBT test will then be locked down and validated in Phase 2 on 250 participants per site.

**Discussion:**

By targeting confirmatory TB testing to those with a positive triage test, 75% of negative GXPU may be avoided, thereby reducing diagnostic costs and patient losses during the care cascade. This study builds on previous biomarker research and aims to identify a point-of-care test meeting or exceeding the minimum World Health Organisation target product profile of a 90% sensitivity and 70% specificity. Streamlining TB testing by identifying individuals with a high likelihood of TB should improve TB resources use and, in so doing, improve TB care.

**Trial registration:**

NCT04232618 (clinicaltrials.gov) Date of registration: 16 January 2020.

## Background

Pulmonary tuberculosis (PTB) is still a leading cause of death and places severe pressure on health care systems of low- and middle-income countries [1]. The number of TB cases is not dropping fast enough to reach the 2030 End TB Strategy milestones and the COVID-19 pandemic has further slowed progress [1, 2]. Each year, 3.6 million people with TB still go undiagnosed, and approximately 30% of patients diagnosed with TB are not treated [2]. The high burden of undiagnosed and untreated TB further fuels ongoing transmission [1–3].

TB testing remains costly, imperfect, and of limited accessibility even in high TB prevalence areas. The highly sensitive and specific GeneXpert® MTB/RIF or Ultra (GeneXpert®) [4] has shown great promise for rapid detection of active TB, but the cost is high. Furthermore, GeneXpert® is in practice frequently a central laboratory-based platform rather than point-of-care (POC), and is subject to logistical challenges and loss to follow-up [5]. Liquid culture is even less accessible than GeneXpert®, prone to contamination, and takes 42 days for a negative result [6]. Traditional chest X-rays (CXR) are also unsuitable as rapid diagnostic or screening tools. They are relatively expensive, non-specific and at present, depend on skilled personnel for interpretation.

Active pulmonary TB will only be confirmed in approximately one-third of those that undergo costly and time-consuming testing for TB based on suggestive respiratory symptoms [7]. This represents an inefficient use of sparse and expensive resources. Focussing TB investigation could streamline resource use, thereby improving TB care in overburdened health care systems [8]. The World Health Organisation (WHO) has re-emphasised the need for efficient TB screening and seeks a non-sputum based screening tool to narrow TB testing to those patients with a high likelihood of PTB [8]. Such a test should be appropriate for global use and have comparable validity to the WHO minimum target product profile (TPP) sensitivity of 90% and specificity of 70% [8].

Specific host biomarkers (human proteins and RNA) have been linked with active TB disease [9–16]. These represent attractive targets for integration into non-sputum-based triage tests [8]. Over a series of previous EDCTP-funded studies since 2010, the Triage Consortium have discovered protein biomarkers present in the blood and associated with a high likelihood of active TB disease [7, 11, 17–19]. These have been progressively narrowed to a signature consisting of three biomarkers (CRP, SAA and IP-10). This signature has been preliminarily validated using samples from African people with TB symptoms (unpublished). Further testing is required to see if this signature would achieve global applicability. As it was validated using serum, this signature also needs further evaluation of its validity on fingerstick blood. A further 10–15 protein biomarkers showed promise for the purpose of triaging symptomatic patients, and further analysis of these is needed to yield the smallest, most practical signature [7, 17].

Subsequent to previous EDCTP-funded studies (AE-TBC and Screen-TB), we advance a prototype lateral flow (LF) based quantitative diagnostic to a point-of-care (POC) applicable test to measure multiple TB-associated host protein biomarkers in fingerstick blood [7, 20, 21]. This multi-biomarker test (MBT) assay comprises a single LF-based test device which measures individual concentrations of multiple biomarkers, using a luminescent label [up-converting reporter particle (UCP) technology] for quantitation. MBT results can be assessed on a battery-powered stand-alone portable reader to yield immediate results. The test format is highly flexible in the number and identity of biomarkers that can be analysed. The UCP label is not hampered by the red colour produced by haemolysis of erythrocytes, allowing convenient dilution of collected fingerstick blood using a lysis buffer. In comparison to other fluorescent labels, the UCP reporter is not light sensitive, does not fade and has an infinite lifetime. The device has been tested in a laboratory environment but has had limited field testing.

### Aims

The TriageTB Study primarily aims to validate, field test and refine the MBT test using fingerstick blood, as well as the Cepheid Xpert® TB Fingerstick (Xpert® TB FS), which is based on host RNA expression.

### Objectives

1. Evaluate promising host protein markers on serum samples from various international sites stored at the Foundation of Innovative Diagnostics (FIND) Biorepository (Phase 1a).
2. Evaluate the MBT strip on serum samples from various international sites stored at the FIND Biorepository (Phase 1a).
3. Enrol participants with symptoms suggestive of active pulmonary TB, perform composite diagnostic workup and classify according to clinical TB status at four international sites. 200 participants during phase 1b and 1000 participants in phase 2.
4. Compare lysed and un-lysed preparation methods for fingerstick blood, and refine the technical format of the MBT test (phase1b).
5. Perform MBT and Xpert® TB FS on fresh fingerstick blood from enrolled participants and compare to gold standard clinical classifications to assess diagnostic accuracy (phase 1b and 2).
6. Store specimens and Chest X-ray images for assessment of accuracy of other diagnostic test candidates, including validation of six transcriptomic signatures which have been associated with active TB (phase1b and 2).
7. Propose combined testing algorithms for efficient TB diagnosis using different modalities.
8. Follow-up participants with confirmed TB to determine treatment outcomes and to collect additional samples for treatment response test discovery and validation (phase 1b and 2).
9. Build capacity by establishing of mentor–mentee partnerships.

## Methods

### Study design

**Fig. 1:**
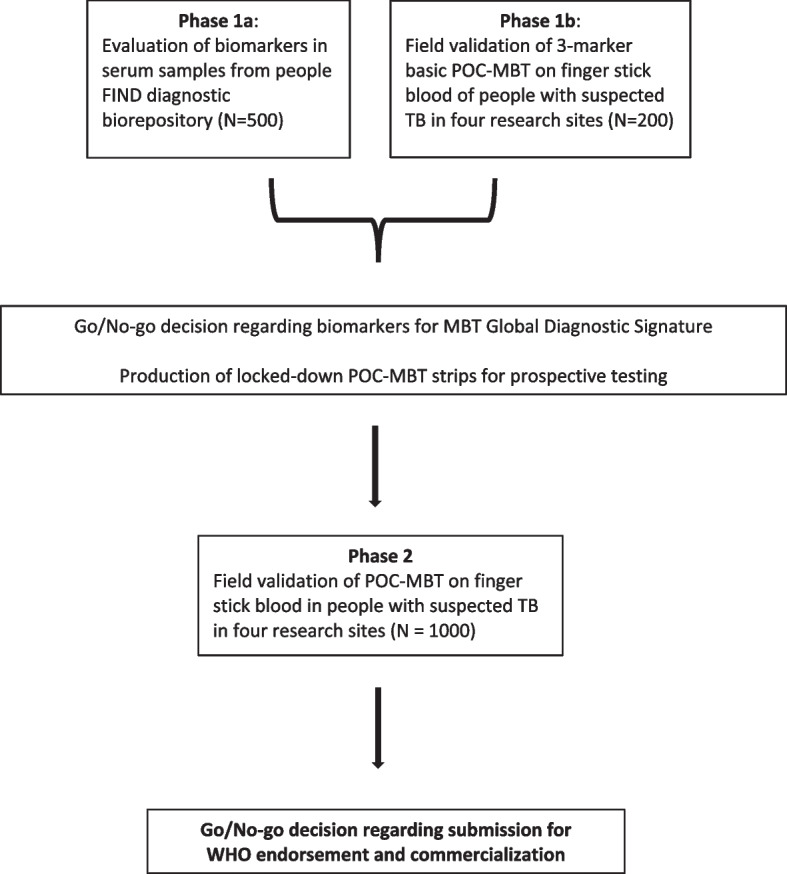
Schematic of study design

This is an observational diagnostic study which will be undertaken in four research sites in countries with high TB prevalence: Stellenbosch University Bio-Medical Research Institute (SU BMRI), South Africa (Clinical site as well as Sponsor of study); Makerere University School of Medicine, Uganda; MRC Unit, the Gambia at London School of Hygiene and Tropical Medicine, Banjul, The Gambia; and Oxford University Clinical Research Unit, Vietnam. Phase 1a and 1b focus on refining the host protein biosignature and field testing the MBT for refinement and lockdown. Phase 2 focuses on validating the final MBT (Fig. 1).

During Phase 1a, sensitivity and specificity for active TB will be determined for serum biomarkers (CRP, IP-10, SAA, ferritin and 10–15 other promising candidate biomarkers) using 500 stored serum samples obtained through FIND from diagnostic studies in South Africa, Vietnam and Peru using Luminex and Meso Scale Diagnostics (MSD). Phase 1b will validate the MBT among 50 participants per site (total 200), prior to lockdown of applied host biomarkers and test format. Following Phase 1 will be a break in enrolment for analysis and derivation of a global diagnostic biomarker signature to be incorporated into the MBT for Phase 2. Phase 2 will validate the MBT with the final signature among 250 participants per site (total 1000).

The two consecutive phases of the validation study follow the same study schedule. A cross-sectional validation of the POC diagnostics will be followed by a longitudinal evaluation of participants diagnosed with TB, to evaluate the tests’ ability to monitor treatment response and predict cure at the end of treatment. Samples, data and chest X-ray’s from both phases will be stored at the sponsor site’s biorepository and central server respectively to allow for later evaluation of future candidate tests.

### Eligibility

Phases 1b and 2 have the same eligibility criteria. The study sites will recruit from regional healthcare facilities. Participants who have had previous TB, extra-pulmonary TB in addition to pulmonary TB, drug resistance detected on GeneXpert® Ultra or culture, or other concomitant diseases will be included. People living with, and without, Human Immune-deficiency Virus (HIV) will be enrolled. Written informed consent will be obtained from the participant or parent/guardian and assent will be obtained for minors prior to all procedures.

### Inclusion criteria

1. Aged 12 to 70 years.
2. Symptoms suggestive of TB disease: cough for ≥ two weeks plus at least one of the following: fever, malaise, weight loss, night sweats, haemoptysis, chest pain or loss of appetite.
3. Appropriate consent ± assent provided.

### Exclusion criteria

1. Stable permanent residence in study area for less than 3 months; no permanent address or planned relocation in the next six months.
2. Pregnancy or breastfeeding.
3. Hb < 9 g/l.
4. Current systemic steroid use or immune suppression therapy in the past four weeks.
5. On TB treatment or Isoniazid Preventive Treatment (IPT) currently or in the last ninety days.
6. Quinolone or aminoglycoside antibiotic use in the past 60 days.
7. In such circumstances where investigator judges there to be a problem with the validity of the consent (e.g. because of suspected mental impairment) or with completion of study procedures (e.g. because of substance abuse).

### Study schedule

On enrolment, participants will receive a standard TB work-up, consisting of clinical assessment, chest X-ray, and sputum GeneXpert® Ultra, smear and culture for *Mycobacterium tuberculosis* (Mtb). Investigational tests will include fingerstick blood testing with the MBT and Xpert® TB FS; stored urine for future use; serum from venous blood for analysis of additional biomarker signatures using Luminex; and whole blood PAXgene tubes for transcriptomic analysis by Fluidigm.

Participants who test positive for TB will follow the study schedule shown in Table 1. As soon as the diagnosis is made, participants will be recalled to TB Day 0 visit for counselling and referral to the local treatment facility to make the final treatment decision and provide TB care. Participants started on standard 6-month TB treatment at clinics will be followed up for clinical review at weeks 4, 8, and 16, at month 6 for end of treatment evaluation, and then at months 12 and 18 to review for possible recurrence. For confirmed TB relapses, Mtb genotyping of baseline and relapse visits will differentiate between relapse and reinfection.

**Table 1:** Study schedule for participants with active TB (Phase 1 and Phase 2)

	**Screening/ Enrolment**	**Day 0**^**a**^	**Week** **4**	**Week** **8**	**Week** **16**	**Month** **6**	**Unscheduled/** **TB Relapse 1**	**Month** **12**^c^	**Unscheduled/** **TB Relapse 2**	**Month** **18**
Informed consent/assent	X									
Eligibility criteria	X									
Medical history	X	X	X	X	X	X	X	X	X	X
TB symptoms	X	X	X	X	X	X	X	X	X	X
POC safety tests	X	ICI					ICI		ICI	
HIV-1 testing	X									
Vital signs/directed physical exam	X	X	X	X	X	X	X	X	X	X
Blood sample	X	X	X	X	X	X				
POC FSB tests	X			X	X	X				
Urine	X			X	X	X				
Sputum	X			X^b^	X^b^	X^b^	ICI	ICI	ICI	ICI
Nasopharyngeal swab	X						ICI		ICI	
Chest X-ray	X			ICI		ICI	ICI	ICI	ICI	ICI
Further care provision/referral	ICI	X	ICI	ICI	ICI	ICI	ICI	ICI	ICI	ICI

*FSB* Fingerstick blood, *HIV* Human immune-deficiency virus, *ICI* If clinically indicated, *POC* Point-of-care

^a^TB call-Back

^b^MGIT culture only

^c^Phone-call ± visit if clinically indicated

Participants who do not test positive for TB at screening will be followed up after 8 weeks for clinical review, further testing if required and/or referral to appropriate facilities (Table 2). An unscheduled visit may be conducted before or after the routine week 8 visit for clinical review or repeat investigation.

**Table 2:** Study schedule for TB-negative participants (Phase 1 and 2)

	**Screening / Enrolment**	**Unscheduled visit**^**a**^	**Week 8**	**Unscheduled visit**^**a**^
Informed consent/assent	X			
Eligibility criteria	X			
Medical history	X			
TB symptoms	X	ICI	X	ICI
POC safety tests	X	ICI		ICI
HIV-1 testing	X			
Vital signs/directed physical exam	X	ICI	X	ICI
POC fingerstick blood tests	X			
Blood	X			
Sputum	X	ICI	ICI^b^	ICI
Urine	X			
Nasopharyngeal swab	X			
Chest X-ray	X	ICI	ICI^b^	ICI
Provision or referral for further care	ICI	ICI	ICI	ICI

*HIV* Human immune-deficiency virus, *ICI* If clinically indicated, *POC* Point-of-care

^a^An unscheduled visit may be done before or after the Week 8 visit to allow for informing participants of notable results or findings, clinical follow-up ± appropriate referral, repeat or additional investigations as indicated. The decision to complete an unscheduled visit will be at the clinicians’ discretion

^b^Investigations indicated if TB diagnosis is still uncertain at this stage or to evaluate response to treatment received

### MBT format

**Fig. 2:**
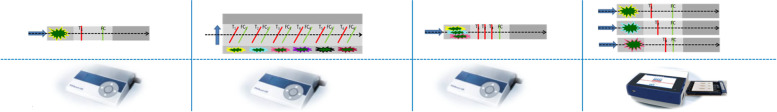
UCP-LF formats. *Figure legend:* Various UCP-LF multi-biomarker test (MBT) formats with distinctive biomarker signatures have been built and evaluated in previous studies [21]. While the structure was initially limited by restrictions of the portable UCP-strip readers, the introduction of a portable reader capable of 2D scan (ESE Quant LR3 version) for use in the TriageTB study allowed the application of the parallel MBT format. The use of parallel strips, each specific for a single biomarker, minimizes potential cross reactivity and each strip has standard dimensions, important for production. Available standard cassettes can hold 3 parallel strips. In this study, for CRP, IP-10 and SAA1

**Fig. 3:**
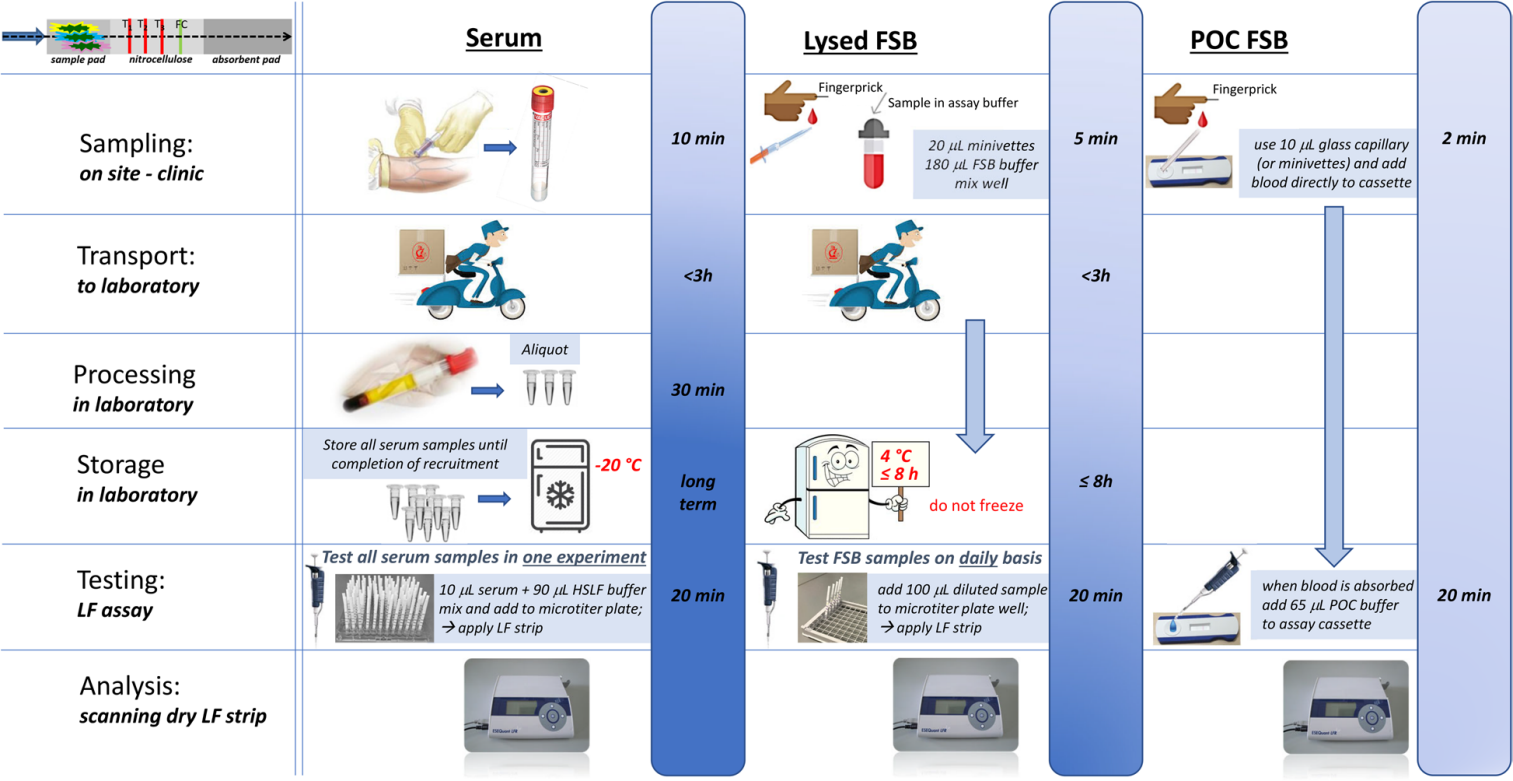
Evaluation of fingerstick blood application. *Figure legend:* During Phase 1B of Triage the basic 3-marker MBT strip with CRP, IP-10 and SAA1 will be evaluated and compared to serum. The application of lysed and whole FSB will be compared. For the whole fingerstick blood testing strips were provided in a cassette and run horizontally immediately after sampling; for the lysed fingerstick blood and serum testing strips were provided with microtiter plates to run vertically in the laboratory. The ESE Quant LR3 version will be distributed to all sites for reading

Figure 2 displays various available MBT formats. The MBT device can hold various sized lateral flow strips, on which each test line detects and quantifies a unique biomarker. One or more standard-sized strips, each with up to three test lines, or one non-standard-sized strip with width determined by the number of parallel slanted test lines, can be used. Standard size would be preferred when considering low cost production utilizing existing production lines from external manufacturers. The ESEQuant LR3 reader (DIALUNOX GmbH, Stockach, Germany) allows for 2D scanning, which has enabled the parallel MBT format. The use of parallel strips, each specific for a single biomarker, minimizes potential cross reactivity. Available standard cassettes can hold 3 parallel strips with standard dimensions, important for industrial production. In phase 1a of the Triage-TB study, performance of the three markers in the Triage Consortium biosignature will be evaluated on a global cohort, using 3-marker MBT strip formats better suited for potential future production. In Phase 1b of Triage-TB, performance of the MBT on fingerstick blood (FSB) will be evaluated and compared to serum. In addition, lysed FSB will be compared to whole FSB. Testing strips for the whole FSB will be provided in a cassette and run horizontally immediately after sampling. Strips for the lysed FSB and serum testing will be provided with microtiter plates to run vertically in the laboratory. A schematic for the approach followed in phase 1B is shown in Fig. 3. For phase 2 of Triage-TB, the chosen MBT format will need to be suitable for industrialisation and for the implementation of the best performing marker combination.

### Gold standard classification

**Fig. 4:**
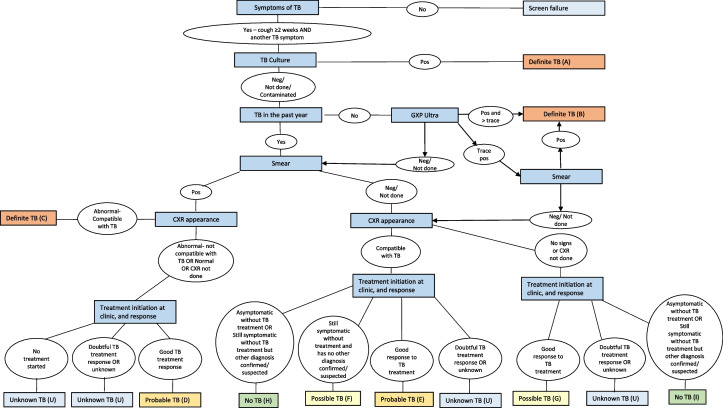
Composite Outcome Classification Algorithm. *Figure Legend*: The gold standard composite diagnostic algorithm comprises symptoms, CXR findings, GeneXpert® Ultra, TB microscopy, TB culture results and treatment response

The POC investigational assays will be measured against a composite diagnostic algorithm comprising symptoms, chest Xray findings, sputum GeneXpert® Ultra, TB microscopy, TB culture results, and treatment response (Fig. 4). Potential outcomes are *Definite TB, Probable TB, Possible TB,* and *No TB.* Participants classified as Definite TB or Probable TB will be regarded as gold standard *positive,* and participants with No TB will be regarded as gold standard *negative*. *Definite TB* denotes sputum culture or GeneXpert® Ultra confirmation; *Probable TB* is a combination of radiological or microbiological evidence with a good response to treatment; and *No TB* indicates that all tests are negative for TB and that an alternative diagnosis exists. *Possible TB* occurs in cases with contradicting evidence, often due to loss to follow-up. This category is excluded from analysis because of the uncertainty of TB disease.

#### Data management and statistics

Data will be entered directly or from paper-source into electronic format using Research Electronic Data Capture (REDCap®). Quality control will include two-step data verification of all forms of data using REDCap® in-built QC capability. The database will also undergo regular central monitoring of key fields by the Sponsor to ensure completeness and accuracy. Classification into gold standard TB Outcome groups will be automated and verified by the attending clinician. Data analysis will be performed by the Stellenbosch University Bioinformatics unit. Basic descriptive statistics will be used to describe the study population. All estimates will be reported with 95% confidence intervals (CI). The main outcomes of interest are the sensitivity, specificity, positive and negative predictive values of the MBT, biomarkers and biosignatures, and algorithmic combinations to differentiate *Definite* and *Probable TB* from *No TB*. These will be analysed using Receiver Operating Characteristic (ROC) curves with Area Under Curve (AUC). There will be a direct comparison of biomarker quantification using the 3-marker MBT and Luminex in Phase 1, determining the reproducibility of the laboratory-based tests and POC tests, using the Kappa statistic for agreement, as well as the performance of the tests in different countries and sub-populations such as those with HIV, previous TB episodes and smear-negative TB. To arrive at the global diagnostic signature, predictive algorithms will be developed using machine learning methods and tested for out-of-sample performance. These will be adapted if the bio-signature requires amendment.

Sample size calculations are for the validation of the MBT in Phase 2, required endpoints being sensitivity, specificity, positive and negative predictive value with 95% CI, with half-width dependent on sample size. For Phase 2, 80 participants (one-third of the 250 per site) are expected to have active TB from previous trial experience, resulting in a target sensitivity of 90% with a 95% CI half-width of 5%. Thus, target precision should be achieved for analysis of MBT performance at each site and overall.

## Discussion

The Triage-TB study will field-test biomarker-based POC triage tests for active TB, in the pursuit of a streamlined workflow that reduces the cost of unnecessary TB testing and takes place where the patient first presents. The Triage Consortium also aims to expand global research on biomarkers associated with active TB.

The great strength of this study is the representation of countries from Africa and Asia in the biomarker evaluation, which allows the investigators to determine a diagnostic signature with true global relevance. In addition, the robust composite diagnostic TB algorithm used in Triage-TB to assign the gold-standard TB classification, was developed and extensively refined during previous Consortium studies (AE-TBC, ScreenTB, PredictTB). Lastly, the inclusion of adolescents (ages 12 to 18 years) in Triage-TB allows the team to investigate whether the biosignatures which were originally developed in adults are also applicable to this age group. A limitation of the study is the current lack of generalisability of the results to children younger than 12, where TB diagnosis can be challenging, and would benefit from a non-sputum based triage test. Another Triage Consortium study, EnDx ChildX, will evaluate biomarkers in children under 12-years old.

The ultimate goal of the TriageTB study is to produce a highly sensitive, “rule-out” test for active TB that is laboratory-free, low-cost, easy-to-use, inexpensive and useable globally. By streamlining the TB diagnostic process there will be a more productive and economical use of valuable TB diagnostics allowing for redirection of resources to those with active TB.

## Data Availability

Not applicable.

## Abbreviations

AUC: Area under curve
CI: Confidence intervals
EDCTP: The European & Developing Countries Clinical Trials Partnership
HIV: Human Immune-deficiency virus
IPT: Isoniazid preventative treatment
MBT: Multi-biomarker test
MTB-HR: Mycobacterium tuberculosis host response
POC: Point-of-care
PTB: Pulmonary tuberculosis
REDCAP: Research electronic data capture
ROC: Receiver operating characteristics
TB: Tuberculosis
u-LAM: Urinary lipoarabinomannase
WHO: World Health Organisation
